# ‘My Data’ project: Understanding the perceptions of administrative data usage for research of people who use drugs

**DOI:** 10.23889/ijpds.v8i2.2259

**Published:** 2023-09-14

**Authors:** Louise Marryat, Camila Biazus-Dalcin, Hazel Booth, Sarah Gray, Joan Love, Andrea Mohan, Sreekanth Thekkumkara

**Affiliations:** 1 University of Dundee, Dundee, United Kingdom

## Background

Administrative data research requires trust that data will be used sensitively and wisely. People who use drugs are frequently stigmatised, and trust may be a particular barrier. This project aims to understand the perceptions of people who use drugs around the use of their administrative health data for research purposes.

## Methods

This project will work with Restoration Fife, a third-sector organisation based in Fife, Scotland, that supports people who use drugs. We are conducting focus groups exploring how administrative health data are used in research from the perspectives of people who use drugs, including discussion around different types/sources of data. Data will be analysed using the Framework approach. We will also work with an artist and members of Restoration Fife, to co-produce a short, animated film about how administrative data are used in research around drug use, in order to educate the wider population about how their data are used.

## Results*

This presentation will discuss findings from the focus groups on the perceptions of usage of administrative data for different types of research. It will also discuss the use of administrative data in the context of findings from previous studies involving general populations and populations with other vulnerabilities, such as care-experienced populations and people with mental health difficulties. We will also provide a viewing of the film within this paper session.

*This project is funded by Research Data Scotland and runs from April to September 2023: all results will therefore be available by the time of the ADR conference in November 2023.

## Conclusion

Evidence suggests low levels of public awareness of how and why data are used. We know little about perceptions of people who use drugs, for whom trust of services may be a particular issue. This study uses innovative methods to provide a platform for voices rarely heard in this context.

